# Perfluoroalkyl Substances (PFASs) in the Canadian Freshwater Environment

**DOI:** 10.1007/s00244-022-00922-x

**Published:** 2022-03-26

**Authors:** Benoit Lalonde, Christine Garron

**Affiliations:** Water Quality Monitoring and Surveillance Division, Water Science and Technology, Environment and Climate Change Canada, 45 Alderney Drive, Dartmouth, NS B2Y 2N6 Canada

## Abstract

Per- and polyfluoroalkyl substances (PFASs) are anthropogenic substances that are very stable in the receiving environment. Legacy perfluoroalkane sulfonates (PFSAs) and perfluoroalkyl carboxylic acids (PFCAs) are especially persistent and resistant to typical environmental degradation processes and therefore are distributed across all trophic levels and environmental compartments (soil, air, water). Since most uses of perfluorooctane sulfonate (PFOS), perfluorooctanoic acid (PFOA), and LC-long-chain PFCAs are banned in Canada, alternative PFASs have been in use for a number of years. Twenty-nine sites across Canada were sampled for PFASs to determine concentrations and trends. Overall, 13 PFASs were measured in 566 Canadian freshwater samples from 2013 to 2020 with a range from below the detection limit (LOD range: 0.4–1.6 ng/L) of the laboratory to a maximum of 138 ng/L (for PFBS). While PFOS and PFOA concentrations are declining significantly over time, other compounds such as PFPeA and PFBA have increased significantly over 2013–2020. Overall, the range of concentrations found in this study was similar to that of other Canadian and international studies. However, this study also found a higher frequency of detections of the replacement PFASs than that of the other, older, Canadian studies.

**Supplementary Information:**

The online version contains supplementary material available at 10.1007/s00244-022-00922-x.

Per- and polyfluoroalkyl substances (PFASs) are anthropogenic substances that may be very stable in the receiving environment. Due to their chemical structures, some of these fluorinated compounds repel both water and oil. They are used extensively in consumer products and industrial applications such as treatments on carpets and clothing, coatings on paper, cardboards and non-stick coatings on cookware, industrial surfactants and emulsifiers and firefighting foams (OECD 2013). Since these products are fabricated, their release to the environment is from industrial manufacturing and the use and/or disposal of PFAS-containing products. Some PFASs are also formed by environmental degradation of related PFSA or precursor compounds (OECD 2013; ATSDR 2018).

Most PFASs are persistent and resistant to typical environmental degradation processes and therefore are distributed across all trophic levels and environmental compartments (soil, air, and water). Although most PFASs are not considered volatile, they readily associate with particulates and may contribute PFASs to surface water through precipitation (OCED 2013; Gewurtz et al. 2019) and have high long-range transport potential (OECD 2021). PFASs as a group contain more than 4700 substances with perfluorooctane sulfonate (PFOS) and perfluorooctanoic acid (PFOA) the two most frequently measured. They are two main types of PFASs: long and short chain with long chains defined as having six or more perfluoroalkyl carbons (OECD 2021). The long-chain PFASs include such compounds as PFOS, PFOA, PFNA, and PFHxS. The length of the carbon chain may result in different bioaccumulative, toxicity and persistence tendencies in the environment (OECD 2021). Myers et al. (2012) depicted short-chain PFASs as more common in urban and industrial watersheds.

In the USA, the longer-chain PFASs (PFOS and PFOA) have historically been produced in the largest quantities (ATSDR 2018) and were both phased out in part due to the groundwater contamination affecting millions of its citizens (EPA 2017). Risk reduction measures on PFOA/PFOS including bans are in place throughout the world (China, Japan, Korea, Sweden, Russia, Australia, etc.) (OECD 2021). The Canadian government has deemed PFOS, PFOA and LC-PFCAs, their salts and precursors toxic to the environment, persistent and bioaccumulative (Longpré et al. 2020). Furthermore, PFOS, PFOA and LC-PFCAs (perfluorocarboxylic acids) (and their salts and precursors) are also prohibited from manufacture, use, sale, offer for sale or import (GOC 2016). More recent scientific evidence indicates that some replacement PFASs, which are being used in place of the already prohibited compounds, may also be associated with adverse environmental effects. These replacement PFASs include such compounds as PFBA, PFBS, PFPeA, PFHxA and PFHpA and are shorter-chain compounds than their predecessors. Nevertheless, Longpré et al. (2020) and Gewurtz et al. (2019) suggested that current and past uses of any PFAS-containing products, as well as long-range transport deposition, may continue to contribute those substances the Canadian environment. In fact, Goodrow et al. (2020) detected those shorter-chain PFASs in the majority of surface water samples in the USA. Similarly, Munoz et al. (2015) measured PFASs in 133 French rivers and lakes with the majority of high concentrations detected near large urban and industrial areas. Nation-wide efforts in Korea and Sweden have also yielded point and diffuse sources of PFAS (Lam et al. 2014 and Nguyen et al. 2017, respectively). The meta-analysis by Kurwadkar et al. (2021) of the presence and levels of PFASs in surface waters (and other media) demonstrates that PFASs are ubiquitous throughout the world.

The objectives of this project were to assess the frequency of detection of PFASs in Canadian freshwaters, to quantify their concentrations and to assess trends over time of both long- and short-chain PFASs. This PFAS monitoring work was intended to assess concentrations in a variety of ambient surface waters in the country and not to target specific releases from industrial sources.

## Methods

### Monitoring Strategy

**Fig. 1:**
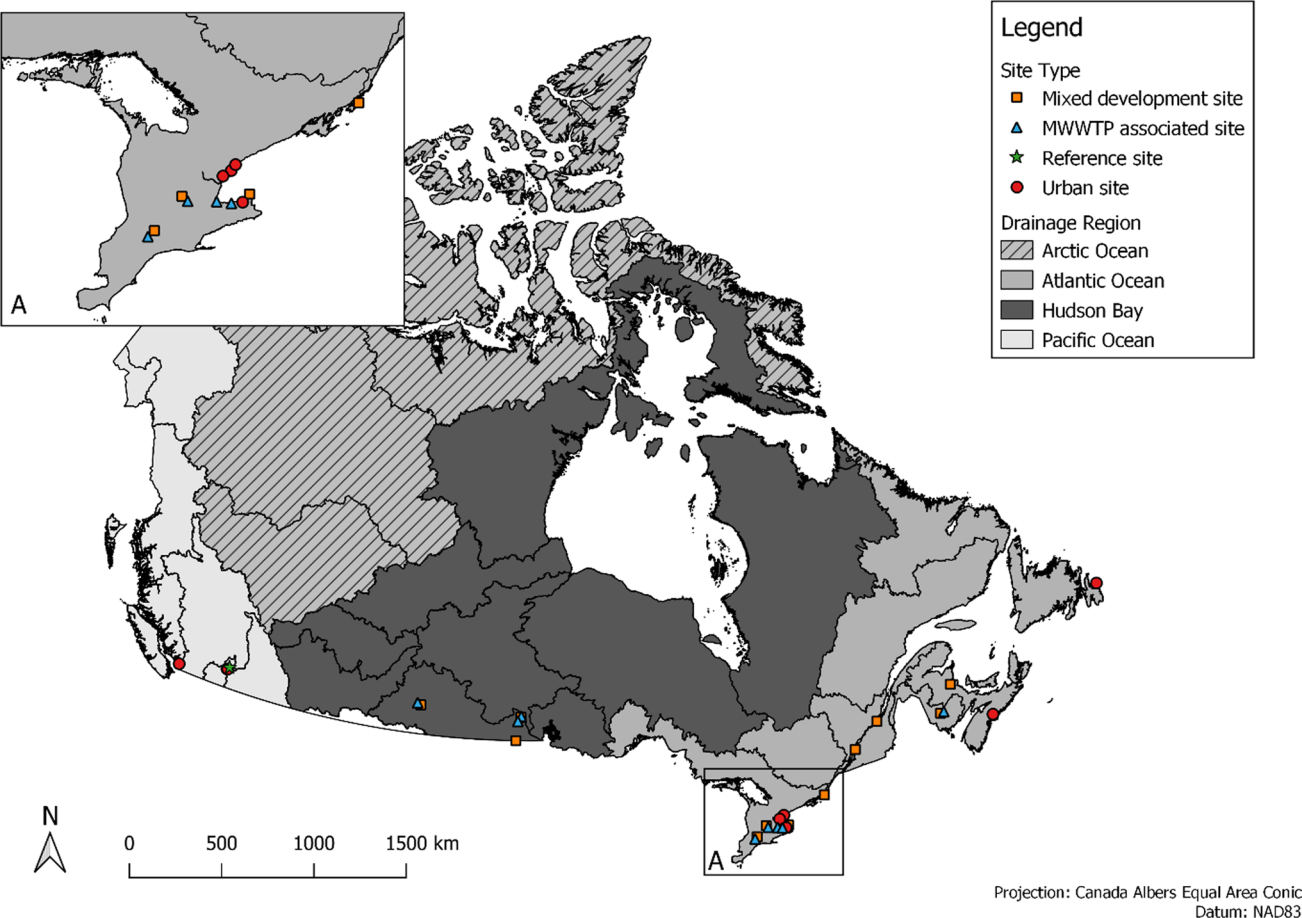
PFASs sampling locations within each ocean area watershed of Canada

Sampling sites for this study were selected to reflect a range of population densities across Canada, and a focus was on areas where PFASs would be released from into the environment (Table 1 in Supplemental Information and Fig. 1). Twenty-nine sampling sites across Canada were sampled between 2013 and 2020. Sampling sites were categorized into four groupings based on dominant activities occurring upstream in the watershed: reference, mixed development (mixture of urban, agricultural and/or forested areas), urban and municipal wastewater treatments plants (MWWTP)-associated sites [Table 1 in supplementary information (SI)]. Where sampling was associated with municipal wastewater effluent discharges, they were not collected at end of pipe, nor within the mixing zone. Those sites were well downstream of MWWTPs and represent ambient surface water conditions in areas that are influenced by wastewater discharges. The reference category only contained one site, mixed use had 11 sites, and urban and MWWTP-associated had nine and eight sites, respectively (Table 1 in SI). The reference site was chosen in an area devoid of all human disturbances except for atmospheric transport of contaminants.

**Table 1:** Summary statistics of the PFAS compounds measured in Canadian freshwater sites

Compound [LOD (ng/L)]	Number of detections	% censored	Maximum (ng/L)	Median (ng/L)	Standard deviation (ng/L)
PFAS compounds with high detection frequencies					
PFBA (1.6 ng/L)	378	32	73	2.64	5.4
PFHxA (0.4 ng/L)	375	34	137	1.96	9.3
PFOA (0.4 ng/L)	350	38	24.4	1.52	4.2
PFOS (0.4 ng/L)	325	43	27.6	2.3	5.9
PFHpA (0.4 ng/L)	300	47	32.7	1.16	2.9
PFPeA (0.8 ng/L)	266	53	47.8	1.3	6.4
PFAS compounds with low detection frequencies					
PFHxS (0.4 ng/L)	102	82	24.4	2.5	5.3
PFBS (0.4 ng/L)	80	86	138	2.6	15.8
PFDA (0.4 ng/L)	70	88	10.7	2.2	2.5
PFNA (0.4 ng/L)	69	88	44.6	1.1	5.4
PFOSA (0.4 ng/L)	17	97	1.7	0.9	0.4
PFUnA (0.4 ng/L)	5	99	9.9	1.4	3.8
PFDoA (0.4 ng/L)	1	> 99	1.4	1.4	1.4

### Sample Collection

Surface water samples were collected by wading into the waterbody or by using a sampling pole from the shoreline, while a smaller fraction were obtained from the middle portion of bridges or from a boat. All samples were collected in 1-L contaminant-free (trace clean) wide-mouth plastic bottles, immediately placed in coolers packed with ice and delivered to the analytical laboratory within 24–48 h post-collection. Trace clean bottles were achieved by following EPA cleaning procedures listed in the OSWER Directive 9240.0-05A “Specifications and Guidance for Contaminant-Free Sample Containers” (EPA 1992).

From 2012 to 2020, quality assurance and control samples comprised 12 field blanks and 15 sets of triplicate samples. The field blank consisted of a 1-L plastic bottle filled with ultra-pure water (using Millipore Sigma water purification systems), which was exposed for 20 s on site. Triplicate samples were obtained using a single 4-L amber glass bottle, which was mixed gently and sub-sampled into three 1-L bottles.

### Laboratory Analysis

Laboratory analyses were conducted by AXYS Analytical Services Ltd. in Sidney, BC, according to AXYS method MLA-060 and MLA-110b (AXYS 2020 and 2021), respectively) by liquid chromatography–tandem mass spectrometry (LC–MS/MS).

Water samples were extracted by solid-phase extraction (SPE) using weak anion exchange cartridge (Water Oasis Wax 150 mg) (AXYS 2021). Sample extracts were then treated with carbon powder, spiked with recovery standards (seven individual standards: ^13^C_3_-PFBA, ^13^C_2_-PFHxA, ^13^C_4_-PFOA, ^13^C_5_-PFNA, ^13^C_2_-PFDA, ^18^O_2_-PFHxS and ^13^C_4_-PFOS) and analyzed by LC–MS/MS (AXYS 2021). Sample analysis was performed on an ultrahigh performance liquid chromatography reversed-phase C18 column using a solvent gradient. The column was coupled to a triple quadrupole mass spectrometer run at unit mass resolution in the multiple reaction monitoring (MRM) in negative electrospray ionization mode.

A mid-level calibration was also analyzed after every 12 h (AXYS 2020). Sample concentrations were determined by the isotope dilution/internal standard method quantification procedures (AXYS 2021). Sample detection limit (DL) was determined by converting the area equivalent to 3.0 times the estimated chromatographic noise height to a concentration in the same manner that target peak responses are converted to final concentrations (AXYS 2021). The MS acquisition rate was at least ten data points per peak (AXYS 2020).

Spiked reference samples and laboratory blank samples were also analyzed for each batch of samples collected for quality assurance and quality control issues. Each batch may contain up to 20 samples, one procedural blank and one spiked matrix sample (AXYS 2020). The spiked matrix is PFAS-free reagent water. The SPM was spiked at approximately the mid-level of the calibration. Samples were deemed acceptable if the recovery of the spiked samples ranged from 70 to 130% (AXYS 2020) and laboratory blanks were below the DL of the laboratory (range from < 0.4 to < 1.6 ng/L per sample depending on the compound) (AXYS 2020). Spiked reference samples were processed alongside samples and used to demonstrate ongoing method precision and recovery. All values used in this paper were deemed acceptable against both laboratories quality control specifications. Water samples hold times are 90 days at < 20 °C, dark (AXYS 2021).

The Canadian Association accredits AXYS Analytical Laboratory for Laboratory Accreditation (CALA) to the standard ISO/IEC 17025, and it holds analysis accreditation with the NELAC (National Environmental Laboratory Accreditation) Institute.

### Statistical Treatment

All statistical analyses were produced using Systat™13 and R (2013). Since a high proportion of the dataset of this study were under the DL of the laboratory (censored value) (Table 1), it is important to use statistical tools, which include all values. Helsel (2005) describes how a mere substitution of the censored value by some fraction of the DL (such as ½ DL) has resulted in inadequate summary statistics and may obscure patterns and trends in the data and suggests instead the use of nonparametric methods to describe such datasets. The methods used to analyze this dataset are included in the non-detects and data analysis (NADA and NADA2) user-written package for R. The details of these methods are described in the books by Helsel (2005, 2012, and 2020). The NADA package uses survival analysis methods to estimate descriptive statistics.

Helsel and Lee (2006) suggested that the censored maximum likelihood (MLE) method may be used when the dataset contains more than 50 detected observations to estimate summary statistics such as the median, mean and standard deviation. The medians depicted in Fig. 1 were calculated using MLE in the NADA package. Censored box plots were created with NADA, and the lower portions of the boxplots (below the reporting limit) are estimated using regression or order statistics (ROS) (Helsel 2012). ROS is obtained by calculating a linear regression on the logarithmic of the uncensored data versus their normal scores, and the order statistic of the normal distribution is equal to the rank of the observation where ranking accounts for censored data (Helsel 2012). Censored data were also used to calculate trend lines using the Arritas-Theil-Sen nonparametric regression on log-normalized concentrations of the different PFASs using the cenken function of the NADA package (Helsel 2012).

The type of sampling site (mixed, urban and MWWTP-associated) and watershed areas (Atlantic Ocean, Great Lakes, Hudson Bay, Pacific Ocean) were tested using the “cen1way” command of the NADA package to determine whether PFASs concentration varies depending on the type of waterbody sampled. This nonparametric test does not assume any normal distribution of the data and utilized both the censored and uncensored dataset. A post hoc pairwise comparison using the Peto–Peto test was also conducted to determine which pairs differed significantly.

## Results and Discussion

Overall, 13 different PFAS chemicals were detected in 566 Canadian freshwater samples from 2013 to 2020. Concentration of PFASs ranged from below the DL of the laboratory to a maximum of 138 ng/L (for PFBS, Table 1). The median and standard deviations below were calculated using both censored and uncensored values for those compounds which contained enough detections: PFBA, PFHxA, PFOA, PFOA, PFHpA and PFPeA. The other ten compounds listed in Table 1 only had detection frequencies ranging from < 1 to 18%. Such small frequency of detections does not permit the use of censored methods to calculate summary statistics. Therefore, the median and standard deviations for those compounds only included values reported over the DL of the laboratory.

The PFASs with the highest frequency of detections were PFBA, followed closely by PFHxA and PFOA. PFOS was also detected in 43% of the samples (Table 1), and no samples were above the Canadian water quality guideline for PFOS of 6800 ng/L (ECCC 2018). Similar to the results of our study, MacInnis et al. (2019) also detected a high frequency of detection for PFBA in the high arctic due to atmospheric deposition, while Goodrow et al. (2020) detected PFOA, PFHpA and PFPeA at all sampling sites in New Jersey. Wang et al. (2019) reported PFBA as the dominant compounds detected in urban lakes and rivers in Beijing (China), which is similar to our results where PFBA had the highest detection frequency (Table 1). The study by Gewurtz et al. (2019) also showed detection greater than 30% for the same six PFASs in the Canadian Great Lakes samples although that study also showed higher detections for PFNA, PFDA, PFUnA and PFDoA.

The subsequent result and discussion on temporal and spatial trends will focus on the six PFASs products, which occurred more frequently: PFBA, PFHxA, PFOA, PFOS, PFHpA and PFPeA (Table 1).

### Temporal Analysis

**Fig. 2:**
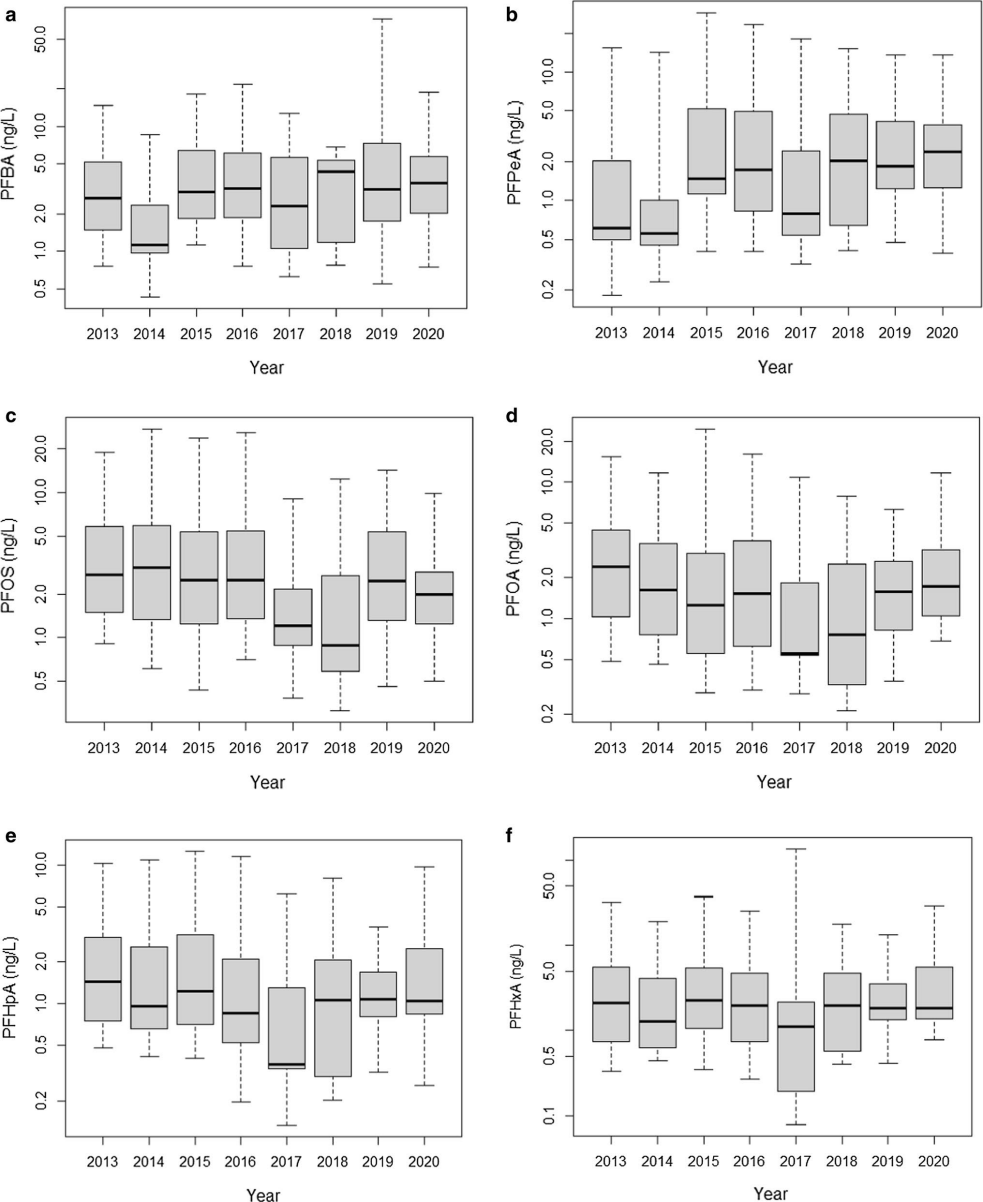
**a**–**f** Boxplots of the most frequently detected main PFASs measured in the Canadian surface waters from 2013 to 2020. **a** PFBA, **b** PFPeA, **c** PFOS, **d** PFOA, **e** PFHpA and **f** PFHxA

Box plots using both censored and uncensored data were created for the six PFASs that had the highest detection frequencies (Fig. 2a–f). The majority of values range from 0.2 to 5 ng/L with a few outliers above 50 ng/L (PFBA, PFHxA, Fig. 2a and f). A visual analysis of the possible increasing or decreasing trends of Fig. 2a–f does not reveal any obvious patterns. However, the dataset contains 8 years of data and therefore is suitable to be analyzed using trend analysis. Figure 2c clearly shows how the maximum value of PFOS in this study is well below the Canadian guideline of 6800 ng/L by two orders of magnitude.

Censored Kendall trends were performed on the six PFASs compounds that had the highest detection frequencies (Table 2). Table 2 presents the results of these trends (slope, intercept tau and *p* values). There were not enough data in each “season” to run any seasonal trend analysis. Apart from PFHxA, the other five compounds all had statistically significant trends although the direction of the trends varied by compounds (Table 2). From the Kendall calculations, PFBA and PFPeA concentrations are increasing significantly in the years of this study, while PFOS, PFOA and PFHpA concentrations are decreasing (Table 2).

**Table 2:** Censored Kendall trends results for PFAS at all sampling stations

Compounds	Slope	Intercept	Tau	*p* value
PFBA	0.205278	− 410.926	0.063596	0.02496
PFHpA	− 0.10458	211.9609	− 0.07095	0.011509
PFHxA	− 0.03689	76.2428	− 0.01523	0.587707
PFOS	− 0.27462	556.0784	− 0.08466	0.002574
PFOA	− 0.165	334.142	− 0.08167	0.003643
PFPeA	0.389608	− 784.015	0.102524	0.000259

The decreases in PFOS and PFOA concentrations are not surprising, as these compounds have been managed extensively in Canada since 2008 and 2010, respectively (ECCC 2021). Furthermore, these substances are also controlled through the Stockholm convention on persistent organic pollutants. Similar decreasing trends in PFOS/PFOA have been reported in other Canadian and international studies (Gewurtz et al. 2019; Nguyen et al. 2019; Land et al. 2018; Hong et al. 2015). As for the significant decline in PFHpA in our study, Kirchgeorg et al. (2013) also measured a significant decrease overtime of this compound in snow from the Alps and noted the decline was probably due to the change from longer- to shorter-chain PFSAs.

The increase in PFBA and PFPeA might be attributed to an increase in the use and disposal of products containing these substances in lieu of the traditional PFOS/PFOA. An increase in PFBA was also detected in Great Lakes samples in the Gewurtz et al. (2019) study, while a significant increase in PFPeA was detected in MWWTP influent in Australia (Nguyen et al. 2019). Morales-McDevitt et al. (2021) also suggest that the prevalence of PFBA in water samples in Bangladesh probably stems from the textile industry’s switch to shorter-chain PFAS alternatives.

Urban streams in China and the USA also contained high detection of PFPeA and PFHxA, and the concentrations were attributed to MWWTP, runoff from land use and landfill and not manufacturing (Moller et al. 2010; Bai and Son 2021; Xu et al. 2021). A meta-analysis of time series of PFASs across all media did not yield enough detections in the studies analyzed for PFBA, PFHxA and PFPeA to determine an overall time series trend (Land et al. 2018).

### Spatial Analysis

Sampling sites and frequencies changed during the course of this study, and this may influence the calculations of the previous trends per compound (Table 2). For example, sampling at mixed-used sites declined in the later years of this study. Careful consideration of the dataset revealed that there were five sites in particular that had been sampled for 6 to 8 years at a frequency ranging from four to seven occurrences per year. A subsequent trend analysis of those five sites [Highland Creek (ON), Red River (at Selkirk, MB), Taylor Creek (ON), Wascana Creek (SK) (downstream of MWWTP) and St. Lawrence River (Qc)] was undertaken and is presented below. Censored Kendall trends were performed on the six PFASs compounds at those five sites, which had the highest frequency of sampling and resulted in 30 individual trend results. Of those 30 trend calculations, only three were statistically significant (Table 2—SI). There were two statistically significant increasing trends for PFPeA at Taylor and Wascana Creek (downstream) and a statistically significant decreasing trend for PFOS at Wascana Creek (downstream) (Table 2—SI).

The site at Red River did not yield any statistically significant trends. However, it is interesting to note that every single sample obtained at this site since 2014 contained a measurable value of PFBA, while the majority of the concentrations reported for the other five main PFASs were below the DL of the laboratory. This also occurs for the St. Lawrence site, which, with the Red River, is the site with the highest discharge volumes/watershed size of this study. This result may be explained by the results from the Gewurtz et al. (2019) study which suggested that wet deposition is contributing to PFBAs in Canadian freshwater.

The St. Lawrence River site had the highest number of observations of all the sites of this study spread over 8 years of sampling. All of the trends calculated were non-significant (Table 2—SI). Comparing the results of this site to that of the other four sites in Table 2—SI, it becomes evident that a vast majority of the samples obtained at this site contained a concentration less than the DL of the laboratory. Interestingly, the substance PFBA that was seldom detected in 2014 and 2015 becomes prevalent in the last few years of sampling at this site. Since PFBAs are both persistent and a short-chain PFAS, it is likely that the increase in concentration might be attributed to the replacement of longer-chain PFOS/PFAS with PFBA. In a 2005–2008 study, Scott et al. (2009) did not measure any concentration of PFBA in 38 Canadian rivers including the very same site on the St. Lawrence River. PFBA was also characterized a potential breakdown product of other in-use PFASs which would also support some our results (MDH 2017a, b). PFBAs are often the PFAS most frequently detected in air samples, snowpack, glacial melt water and surface waters (Kirchgeorg et al. 2013; Kwok et al. 2013; MacInnis et al. 2019; Muir et al. 2019) in the high Arctic which gives indication that PFBAs are uniformly distributed in the northern hemisphere due to long-range transport.

The Highland Creek site did not yield any statistically significant trends. While PFOS and PFAS detections at this site were reported in approximately half of the samples, PFBA, PFHxA and PFPeA were detected in almost all of the samples obtained. Although the upward trend of PFPeA was statistically non-significant (*p* = 0.07), the trend slope shows an increase that should be re-evaluated in the next few years to determine whether the trend becomes statistically significant. The site at Taylor Creek did yield one statistically significant upward trend for PFPeA (*p* = 0.042). Similarly, to Highland Creek the vast majority of samples from this site had detectable concentrations even for PFOS and PFOA although none of the PFSA had a significant trend to report. Urban streams throughout the world have shown high detection frequencies of these PFASs mostly due to runoff from land and not necessarily PFAS manufacturing plants located in the watershed (Goodrow et al. 2020; Bai and Son 2021; Xu et al. 2021).

The Wascana Creek site had two statistically significant trends (Table 2—SI). Of all the sites samples in this study, this is the location where MWWTP discharge influences the natural environment the most. The sampling site on Wascana Creek is located approximately 8.5 km downstream of a MWWTP serving a population of over 190,000 (Table S1). The discharge of this river fluctuates from < 5 to 60 m^3^/s depending on rain, spring freshet. Wascana Creek is known to receive a high proportion of its flow from a wastewater treatment plant during dry spells (Waiser et al. 2011). While it was expected that PFOS concentrations are decreasing over time due to the legislation in Canada, the statistically increasing concentration of PFPeA was novel. While PFPeA concentrations are mostly under the DL of the laboratory from 2013 to 2015, it becomes ubiquitous from 2016 to 2019. This might be a reflection in the change of PFAS compounds in industrial and commercial uses and its ultimate discharge to the MWWTP located upstream of the sampling site.

### PFASs as a Function of Land Use Type

**Fig. 3:**
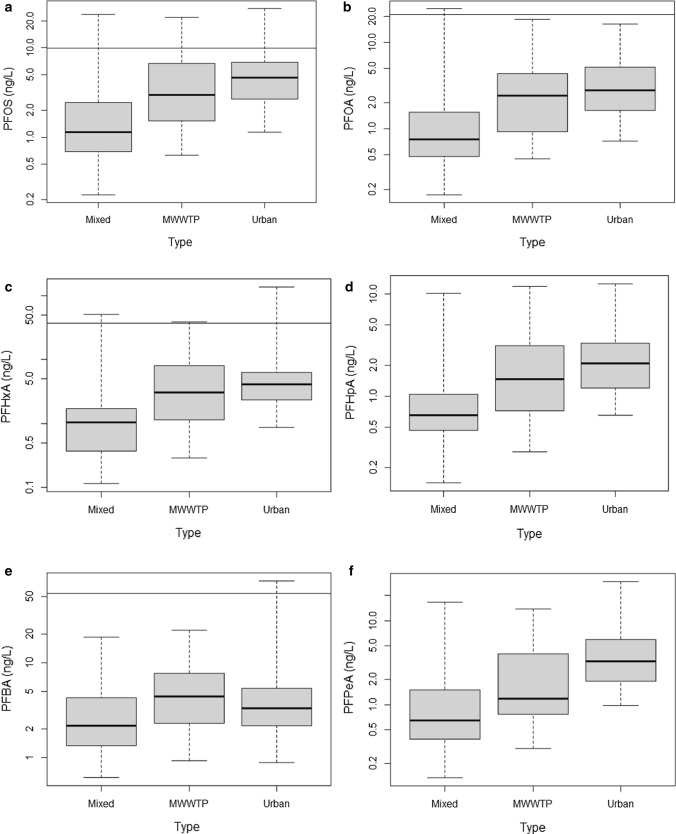
**a–f** Boxplots of the main PFASs measured in the Canadian surface waters as a function of the type of land use type (mixed use, urban and MWWTP associated). The horizontal line represents the highest censoring threshold

All six of the PFAS chemicals had statistically significant different (*p* < 0.05) concentrations between the categories of major land use types. The post hoc pairwise comparison (Peto–Peto) revealed that all pairs for all six PFASs were statistically different from each other. The censored box plots (Fig. 3a–f) reveal an overall trend with five of the six PFASs having the lowest concentrations measured at mixed sites and the highest at urban sites. It is interesting to note that urban waterbodies had significantly higher concentrations than either MWWTP-associated sites or mixed-use sites. This result indicates that most of the PFASs reaching surface waters could be attributed to weathering of material containing these PFASs, urban runoff and wet deposition rather than through wastewater discharge or direct industrial outputs. Similarly, Munoz et al. (2015), Wang et al. (2019) and Bai and Son (2021) detected high frequencies of PFASs in urban streams not always associated with MWWTPs. Recently, Propp et al. (2021) measure significant concentrations of PFAS in landfills across Ontario that had been closed for more than 30 years. This suggests that historic landfill sites may be contributing PFAS to groundwater and possibly to the urban sites sampled during the course of this study.

### Comparison to Other Studies

PFASs have been measured in freshwater systems around the world. Most countries, including Canada, started monitoring for long-chain PFASs such as PFOS/PFOA before refocusing on short-chain PFASs, especially in the last decade. The majority of studies have sampled urban or MWWTP-influenced water bodies to determine the concentrations of PFASs that might be attributed to runoff or industrial wastewaters (Table 3). In addition, there have been many studies conducted in the Arctic to determine the effects of long-range transport of PFASs to an environment where sources of these compounds are minimal if present at all (Table 3).

**Table 3:** Maximum or *mean/median* (bold and italic in table) concentrations of PFASs in surface waters from other Canadian and international studies

Sampling location (no. of samples)	PFBA	PFHxA	PFOA	PFOS	PFHpA	PFPeA	References
Canada							
Great Lakes	4.9	3.9	5	7.4	2	4.1	Gewurtz et al. (2019)
Arctic Lakes	1		< 1		< 0.5		MacInnis et al. (2019)
Lake Ontario (1)			< 3		< 1		Meyer et al. (2011)
Mimico Creek (7)			***14***	***22***	***3.3***		Meyer et al. (2011)
Hamilton Harbour (2)			***7.2***	***13***	***1.4***		Meyer et al. (2011)
Lake Superior (25)		0.8	1.23	0.7			Scott et al. (2010)
WWTP effluent (3)	2.6	6.8	24		3.3	4.1	Scott et al. (2010)
Ontario Rivers (6)		0.18	0.48	0.8	0.42		Scott et al. (2010)
Ontario Rivers (20)		100	52	150	51	150	D’agostino and Mabury (2017)
Grand River (1)		***5.3***	***1.7***	***2.8***	** < ** ***2***	***5.8***	D’agostino and Mabury (2017)
Thames River (1)		***3.9***	***1.4***	***5.7***	** < ** ***2***	** < ** ***5***	D’agostino and Mabury (2017)
USA							
Nevada Brook (8)	nd	56.9	19.2	17.4	10.4	21.8	Bai and Son (2021)
Nevada Brook—downstream of WWTP (10)	26	187	65.5	38	32.5	170	Bai and Son (2021)
Yadkin-Pee Dee River (Carolina) (5)	1.38	4.37	8.07	4.34	5.79	1.93	Penland et al. (2020)
Mississippi; downstream of 3 M facility (1)	650	32	220	136	75		Newsted et al. (2017)
New Jersey River; downstream of WWTP and/or landfill (11)	10	26	33.9	102	14.6	17.7	Goodrow et al. (2020)
Europe							
Surface water—Arctic river (6)	1.2	0.3	0.3	0.29	0.2	1.1	Kwok et al. (2013)
Rhine River (36)	188	4.48	4.07	7.34	0.97	9.99	Moller et al. (2010)
Ruhr River (3)	27.5	13.8	17.9	10.1	1.23	27.6	Moller et al. (2010)
Other German rivers (8)	8.38	9.86	11.7	7.07	1.31	12.1	Moller et al. (2010)
Lake (reference) (20)	1.37	0.13	1.78	0.23	0.42	< 0.01	Skaar et al. (2019)
Arctic Stream near settlement (3)	28.1	61.47	39.28	653	15.43	< 0.02	Skaar et al. (2019)
Asia/Australia							
Influent of a WWTP (9)	19	20	13	17	16	15	Nguyen et al. (2019)
Rivers (7)	8.8	11.4	14.5	45.2	6	9.7	Allinson et al. (2019)
Urban lakes and rivers (350)	75.8	30	69	50.8	11.2	111	Wang et al. (2019)
Surface water—landfill, urban (19)	110	26.24	100.1	54.78	30.3	53.18	Xu et al. (2021)

In Canada, the results from past studies are mostly within the range of concentrations found within our study (Table 3) except for the results from D’Agostino and Maybury (2017). In the D’Agostino and Maybury (2017) study, the maximum concentrations of most compounds are well above those found in this study. However, this may be related to the inclusion of sites knowingly impacted by firefighting foams. It is also interesting to note the ubiquitous measurements of PFPeA, PFHxA and PFBA in this study that were not always detected in past Canadian studies (Table 3). This might be a reflection of new uses of these products in some manufacturing processes. Direct comparison was possible for the following sites: Hamilton Harbour (ON), Mimico Creek (ON), Grand and Thames rivers (ON). In general, PFSA concentrations at the four sites have increased slightly for the majority of compounds. Although, none of the increases were more than an order of magnitude. Both PFHpA and PFHxA saw the largest increases at these four sites while PFOS/PFOA had very similar concentrations in past and present studies. This could be a reflection of the use of new PFASs in the manufacturing process and the persistence of PFOS/PFOA in the environment.

In international studies, the majority of compounds had higher maximum concentrations than that of this study (Table 3). However, some of those international studies were meant to record the effects of industrial outputs directly related to PFASs such as the 3 M facility on the Mississippi River (Newsted et al. 2017) or heavily industrialized waterways such as the River Rhine (Moller et al. 2010) (Table 3). Even some Arctic stream may contain similar PFASs concentrations to that of our study, especially streams located near settlements and/or those influenced by precipitations linked to long-range atmospheric transport (Table 3).

## Conclusion

This study has revealed that PFAS chemicals are found throughout freshwater systems in Canada. The main contributor of PFASs in our study was probably related to urban runoff and secondarily to MWWTPs. Concentrations of older PFASs, such as PFOS and PFOA, are declining, likely due to regulatory measures in Canada and other countries, while some of the shorter-chain PFASs are increasing, probably due to a combination of increased use and long-range transport. Both Myers et al. (2012) and Gewurtz et al. (2019) have suggested that sediment may play a larger role as a continual source of PFASs to surface water. It is suggested that the addition of sediment to surface water monitoring for PFSA would be beneficial to understand the different source of these compounds to the Canadian environment.

## Supplementary Information

Below is the link to the electronic supplementary material.

Supplementary file1 (DOCX 19 kb)

## Data Availability

The datasets generated during and/or analyzed during the current study are available from the corresponding author on reasonable request.
