# Supplementary material for: Perfluoroalkyl Substances (PFASs) in the Canadian Freshwater Environment
Source: Arch Environ Contam Toxicol. 2022 Mar 26;82(4):581–91. doi: 10.1007/s00244-022-00922-x (PMC9079020; doi:10.1007/s00244-022-00922-x)
Supplement: Supplementary file 1 — Supplementary file1 (DOCX 19 kb) [file 244_2022_922_MOESM1_ESM.docx]

**Supplemental Information**

Table 1 SI. List of monitoring sites and geographical and human-related characteristics

| **Site Name (# of samples)** | **Years** | **Latitude (DD)*** | **Longitude (DD)*** | **Watershed population** | **MWWTP**  **(km)** | **River Discharge (m^3^/s)** |
| --- | --- | --- | --- | --- | --- | --- |
| **Mixed Development Sites** | | | | | | |
| Nappan, NB | 2013-14 | 47.030 | -65.384 | 1500 | None | n/a |
| Saint John River, NB (up) | 2013-19 | 45.948 | -66.869 | < 5 000 | 36 | 350- 6 100 |
| St. Lawrence River, QC | 2013-20 | 46.806 | -71.187 | 538 238 | 13 | > 12 000 |
| St. Lawrence River, QC | 2013-20 | 45.875 | -73.281 | 3 824 221 | 29 | > 10 000 |
| St. Lawrence River (Wolfe) , ON | 2013-20 | 44.211 | -76.237 | 11 000 000+ | 90 | >8000 |
| Niagara River, ON | 2013-20 | 43.255 | -79.055 | 12 000 000+ | 15 | >5800 |
| Thames River (up) , ON | 2014-19 | 43.038 | -81.223 | < 40 000 | 33 | 5 - 70 |
| Grand River (up) , ON | 2013-18 | 43.482 | -80.481 | 15 000 | 30 | n/a |
| Red River – Selkirk (up), MB | 2013-18 | 50.141 | -96.869 | 633 450 | 28 | >3200 |
| Red River - Emerson, MB | 2013-20 | 49.001 | -97.223 | 665 | 4.5 | >1 200 |
| Wascana Creek, SK (up) | 2013-20 | 50.416 | -104.549 | 1894 | 14 | n/a |
| **Urban Sites** | | | | | | |
| Waterford River, NFLD | 2013-20 | 47.529 | -52.741 | 24 284 | none | 1.1-3.3 |
| Little Sackville River, NS | 2013-20 | 44.763 | -63.6888 | 21 379 | none | 0.017-8.52 |
| Dicks Creek, ON | 2014-20 | 43.154 | -79.243 | 17 931 | none | n/a |
| Taylor Creek, ON | 2013-20 | 43.701 | -79.312 | n/a | none | n/a |
| Highland Creek, ON | 2013-20 | 43.778 | -79.191 | 360 000 | none | 1 - 40 |
| Mimico Creek, ON | 2013-15 | 43.646 | -79.517 | 155,800 | none | 1-35 |
| Mill Creek, BC | 2013-16 | 49.883 | -119.499 | 117 312 | none | 19.4 |
| Mill Creek, BC | 2013-15 | 49.887 | -119.437 | <117,312 | None | n/a |
| Still Creek, BC | 2013-17 | 49.259 | -122.969 | 100 000 | none | 0.04-0.4 |
| **MWWTP - Associated Sites** | | | | | | |
| Saint John River (dn), NB | 2013-19 | 45.953 | -66.624 | 56 224 | 0.25 | 500 - 7120 |
| Thames River (dn) , ON | 2013-18 | 42.965 | -81.389 | 366 151 | 6 | 10 - >500 |
| Grand River (dn) , ON | 2013-18 | 43.385 | -80.385 | 507 096 | 5 | 25-300 |
| Hamilton Harbour 914 , ON | 2013-20 | 43.268 | -79.781 | >500 000 | 1.5 | n/a |
| Hamilton Harbour 926, ON | 2013-17 | 43.183 | -79.483 | >175 000 | 0.25 | n/a |
| Red River -Selkirk (dn), MB | 2015-20 | 50.191 | -96.844 | 9 934 | 2.75 | >3200 |
| Red River - Winnipeg, MB | 2013-18 | 49.950 | -97.098 | 633 450 | 0.1 | n/a |
| Wascana Creek (dn), SK | 2013-20 | 50.499 | -104.800 | 193 100 | 8.5 | <5 - 60 |
| **Reference site** | | | | | | |
| Mill Creek, BC | 2013-16 | 49.9835 | -119.352 | <50 | none | n/a |

DD decimal degrees, up upstream, dn downstream, *NB* New Brunswick, *QC* Québec, *ON* Ontario, *MB* Manitoba, *NFLD* Newfoundland and Labrador, *NS* Nova Scotia, *BC* British Columbia, *SK* Saskatchewan

Table 2 SI. Censored Kendall trend characteristics for six PFAS compounds at five Canadian surface water sampling sites. Statistically significant correlations (p<0.05) are bolded.

| Sampling Sites | Compound | Slope | Intercept | Tau | p value |
| --- | --- | --- | --- | --- | --- |
| Red River at Selkirk | PFBA | -0.18327 | 375.2694 | -0.05929 | 0.711571 |
|  | PFHpA | -0.5096 | 1028.489 | -0.25 | 0.078364 |
|  | PFHxA | -0.24544 | 496.1067 | -0.13406 | 0.360764 |
|  | PFOA | -0.1329 | 268.9065 | -0.23188 | 0.106955 |
|  | PFOS | 0.135544 | -271.533 | 0.050725 | 0.731914 |
|  | PFPeA | 0.156438 | -314.501 | 0.07971 | 0.583541 |
| Highland Creek | PFBA | 0.191846 | -383.024 | 0.068293 | 0.530338 |
|  | PFHpA | -0.16663 | 338.078 | -0.13178 | 0.213266 |
|  | PFHxA | 0.045318 | -87.4421 | 0.012182 | 0.915997 |
|  | PFOA | -0.14035 | 284.9427 | -0.08749 | 0.409515 |
|  | PFOS | -0.17645 | 359.612 | -0.06202 | 0.562372 |
|  | PFPeA | 0.666124 | -1338.93 | 0.187154 | 0.074069 |
| Taylor Creek | PFBA | 0.252027 | -503.316 | 0.097561 | 0.363177 |
|  | PFHpA | -0.10637 | 216.8658 | -0.11517 | 0.27732 |
|  | PFHxA | -0.17261 | 353.1741 | -0.09524 | 0.37014 |
|  | PFOA | -0.17381 | 354.3083 | -0.13178 | 0.214333 |
|  | PFOS | -0.0609 | 127.797 | -0.02215 | 0.842035 |
|  | PFPeA | 0.776849 | -1562.12 | 0.211517 | **0.042948** |
| St. Lawrence River | PFBA | 0.033984 | -66.9266 | 0.018169 | 0.84992 |
|  | PFHpA | 0.00012 | 0.154691 | 0.000699 | 1 |
|  | PFHxA | -0.18168 | 366.762 | -0.06429 | 0.485057 |
|  | PFOA | -0.04894 | 99.54884 | -0.0559 | 0.543392 |
|  | PFOS | -0.35256 | 711.911 | -0.09364 | 0.303258 |
|  | PFPeA | 0.004873 | -8.86968 | 0.002096 | 0.987729 |
| Wascana Creek (downstream) | PFBA | 0.328056 | -653.903 | 0.070825 | 0.497369 |
|  | PFHpA | -0.02638 | 57.7395 | -0.01212 | 0.914063 |
|  | PFHxA | -0.04768 | 109.1506 | -0.00202 | 0.992165 |
|  | PFOA | -0.54874 | 1111.849 | -0.15556 | 0.133838 |
|  | PFOS | -1.17016 | 2366.181 | -0.25859 | **0.012554** |
|  | PFPeA | 1.768771 | -3561.16 | 0.335354 | **0.00104** |
